# Neural stem cell treatment for perinatal brain injury: A systematic review and meta‐analysis of preclinical studies

**DOI:** 10.1002/sctm.21-0243

**Published:** 2021-09-20

**Authors:** Madeleine J. Smith, Madison Claire Badawy Paton, Michael C. Fahey, Graham Jenkin, Suzanne L. Miller, Megan Finch‐Edmondson, Courtney A. McDonald

**Affiliations:** ^1^ The Ritchie Centre Hudson Institute of Medical Research Clayton Victoria Australia; ^2^ Department of Obstetrics and Gynaecology Monash University Clayton Victoria Australia; ^3^ Cerebral Palsy Alliance Research Institute, Speciality of Child and Adolescent Health, Sydney Medical School, Faculty of Medicine and Health The University of Sydney Sydney New South Wales Australia; ^4^ Department of Paediatrics Monash University Clayton Victoria Australia

**Keywords:** animal models, cell transplantation, immunosuppression, neural differentiation, stem cells, tissue‐specific stem cells

## Abstract

Perinatal brain injury can lead to significant neurological and cognitive deficits and currently no therapies can regenerate the damaged brain. Neural stem cells (NSCs) have the potential to engraft and regenerate damaged brain tissue. The aim of this systematic review was to evaluate the preclinical literature to determine whether NSC administration is more effective than controls in decreasing perinatal brain injury. Controlled interventional studies of NSC therapy using animal models of perinatal brain injury were identified using MEDLINE and Embase. Primary outcomes were brain infarct size, motor, and cognitive function. Data for meta‐analysis were synthesized and expressed as standardized mean difference (SMD) with 95% confidence intervals (CI), using a random effects model. We also reported secondary outcomes including NSC survival, migration, differentiation, and effect on neuroinflammation. Eighteen studies met inclusion criteria. NSC administration decreased infarct size (SMD 1.09; CI: 0.44, 1.74, *P* = .001; *I* ^2^ = 74%) improved motor function measured via the impaired forelimb preference test (SMD 2.27; CI: 0.85, 3.69, *P* = .002; *I* ^2^ = 86%) and the rotarod test (SMD 1.88; CI: 0.09, 3.67, *P* = .04; *I* ^2^ = 95%). Additionally, NSCs improved cognitive function measured via the Morris water maze test (SMD of 2.41; CI: 1.16, 3.66, *P* = .0002; *I* ^2^ = 81%). Preclinical evidence suggests that NSC therapy is promising for the treatment of perinatal brain injury. We have identified key knowledge gaps, including the lack of large animal studies and uncertainty regarding the necessity of immunosuppression for NSC transplantation in neonates. These knowledge gaps should be addressed before NSC treatment can effectively progress to clinical trial.

## Graphical abstract

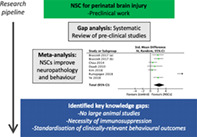

This systematic review and meta‐analysis shows that neural stem cells are a promising treatment for perinatal brain injury. Key knowledge gaps have been identified and should be addressed before the commencement of clinical trials.

**Abbreviations**
bFGF: basic fibroblast growth factor
chABC: chondroitinase ABC
CI: confidence interval
CNPase: 2′,3′‐cyclic‐nucleotide 3′‐phosphodiesterase
Ctip2: chicken ovalbumin upstream promotor transcription factor 2
ESC: embryonic stem cell
FOXP1: Forkhead Box P1
GAD: glutamic acid decarboxylase
GFAP: glial fibrillary acidic protein
HI: hypoxic ischemic
HIE: hypoxic ischemic encephalopathy
Iba‐1: ionized calcium binding adaptor protein 1
IL‐1β: interleukin 1 beta
Inflam: inflammatory
IP: intraperitoneal
iPSCs: induced pluripotent stem cells
KO: knockout
LPS: lipopolysaccharide
MAP‐2: microtubule‐associated protein
MBP: myelin basic protein
MSC: mesenchymal stem cell
n: nimber
NA: not applicable
NDPs: neurosphere derived precursor cells
NeuN: neuronal nuclei
NG2: nerve/glial antigen 2
NPCs: neural progenitor cells
NS: not significant
NSCs: neural stem cells
NSE: neuron‐specific enolase
NSPCs: neural stem progenitor cells
O_2_
: oxygen
Olig2: oligodendrocyte transcription factor 2
OPCs: oligodendrocyte progenitor cells
PBS: phosphate buffered saline
PND: postnatal day
SD: Sprague‐Dawley
SMD: standardarized mean difference
TuJ1+: neuron‐specific class III β‐tubulin
UCBC: umbilical cord blood cell
VEGF: vascular endothelial growth factor
WT: wildtype

**Significance statement**

The limited treatment options for perinatal brain injury and the subsequent life‐long burden define an urgent need to develop neuroregenerative treatments. From preclinical research to date, all performed in rodents, the authors show that neural stem cell administration is an efficacious treatment for perinatal brain injury across neuropathological, motor, and cognitive domains. Before clinical translation of neural stem cell transplantation for perinatal brain injury, important future directions include large animal studies, investigating whether immunosuppression is necessary in the neonate and standardization of clinically‐relevant behavioral outcomes.

## 1 INTRODUCTION

Injury to the developing brain during pregnancy or around the time of birth, termed perinatal brain injury, is a major cause of morbidity and mortality. There are various, complex causes of perinatal brain injury, including inflammation, hypoxia ischemia (HI), excitotoxicity, placental abnormalities and perinatal stroke.^1^ Additionally, fetal growth restriction, chronic hypoxia, infection, and inflammation can render the brain more vulnerable to perinatal injury.^2^, ^3^, ^4^, ^5^ Perinatal brain injury can lead to cerebral palsy (CP), epilepsy, and other permanent neurological disorders.^6^, ^7^, ^8^ Current therapies for many of these conditions are predominantly based on symptom management, including physical rehabilitation and anti‐seizure medication.^9^ Available medical interventions are limited but include therapeutic hypothermia for term‐born infants with neonatal encephalopathy which is associated with significant improvements in neurological function. Unfortunately, half of the neonates treated with hypothermia will still die or have serious adverse neurological outcomes.^10^ Additionally, antenatal corticosteroids and magnesium sulfate provide neuroprotection,^9^ but no treatments are available to repair the underlying brain injury.

Stem cells have been extensively researched to treat neurological conditions as some stem cell types have regenerative, anti‐inflammatory, and neuroprotective properties. Over the last two decades a number of stem/progenitor cell types have been shown to have the capacity to differentiate into neural cell lineages in vitro, including embryonic stem cells (ESCs), induced pluripotent stem cells (iPSCs) and a number of fetal derived stem cells.^11^, ^12^ As such, many stem cell types have been tested and shown potential in preclinical studies to reduce brain injury, including mesenchymal stem cells (MSCs), neural stem cells (NSCs), and umbilical cord blood cells (UCBCs).^12^, ^13^ The mechanisms of action of MSCs and UCBCs for brain injury treatment are primarily trophic and anti‐inflammatory, with no evidence of significant engraftment or neural lineage differentiation.^14^, ^15^, ^16^, ^17^ In contrast, ESCs, iPSCs or NSCs from fetal tissue origin, are anti‐inflammatory and neurotrophic, and they can also engraft into the brain. Here, they can differentiate into the three primary cell types; neurons, astrocytes, and oligodendrocytes^18^ and therefore hold promise to repair and regenerate damaged brain tissue. NSCs can be obtained from a number of sources including fetal or embryonic brain tissue, or they can be differentiated from embryonic stem cells (ESCs) or induced pluripotent stem cells (iPSCs).^19^ NSCs are being investigated to treat adult neurological conditions, including Parkinson's disease, multiple sclerosis, Huntington's disease, spinal cord injury, and stroke.^20^, ^21^, ^22^, ^23^, ^24^, ^25^ Notably, in preclinical models of adult stroke, NSCs significantly reduce brain infarct size and alleviate behavioral deficits,^20^, ^22^ specifically in tests that assess motor function.

NSCs may hold the key to promoting brain repair and are therefore an appealing reparative therapy for perinatal brain injury. There is now a growing body of preclinical evidence investigating the efficacy of NSC therapy; however, there are often conflicting results in the literature. Therefore, we conducted a systematic review and meta‐analysis to determine whether NSC administration is more effective than control/vehicle treatment. The primary outcomes of interest were reduced brain infarct and functional improvement in behavioral (motor and cognitive) tests. Secondary outcomes of interest were NSC survival, migration, and differentiation. Conducting this systematic review may identify preclinical gaps that should be addressed before NSC therapy moves toward clinical trials.

## 2 METHODS

This systematic review and meta‐analysis followed the guidelines of Preferred Reporting Items for Systematic Reviews and Meta‐Analyses (PRISMA, http://www.prisma-statement.org/).^26^ The review protocol was registered on PROSPERO (CRD42021222952).

### 2.1 Selection criteria

Preclinical studies must have utilized a neonatal or perinatal model of hypoxic, ischemic, or inflammatory, or excitotoxic brain injury to be eligible for inclusion. As the main objective of this systematic review was to determine the effectiveness of NSC therapy for perinatal brain injury, all studies must have included a group that were administered non‐transfected or pretreated “neural stem cells,” a term which we have defined to include NSCs, neural progenitor cells (NPC), neurosphere derived precursor cells (NDPCs), neural precursor cells, oligodendrocyte progenitor cells (OPCs), olfactory cells, ensheathing cells, or neuroepithelial cells. For simplicity, we use the term “NSCs” throughout this review to describe all eligible cell types. If a study included an adjuvant or concomitant therapy, it must also have included a non‐transfected or pre‐treated NSC group to be eligible. Cells could be given at any time point following brain injury and via any route of administration. Studies must have included an injured, non‐cell‐treated control group. Eligible studies must have included at least one of the following primary outcomes: brain volume or brain infarct volume; motor function; or cognitive function.

### 2.2 Search strategy

We searched MEDLINE (1946 to September 24, 2020) and Embase (1947 to September 24, 2020) via Ovid using the following strategy: ([neonatal or perinatal or neonate or perinate or newborn].tw) AND ((brain or cerebr* or neuro*) AND (occlusion or stroke or hypoxic or hypoxia or ischemic or ischaemic or ischaemia or ischemia or injury)).tw) AND ([neural stem or NSC* or neuroepithelial or neural progenitor* or NPC* or neuro‐progenitor* or neuro‐epithelial or oligodendrocyte progenitor* or OPC* or olfactory cell* or ensheathing cell* or OEC*].tw) AND ([transplantation or transplant or injection or inject or administration or administer or administered or intracerebral or intranasal or intraperitoneal or intravenous or intravenously or infusion or treatment or treat or treated].tw). Searches were limited to English language articles. To ensure no recent studies were missed, searches were rerun using the same parameters on May 4, 2021.

### 2.3 Study selection process

Deduplicated results from Ovid were exported into EndNote (version X9.3.3). Additional deduplication was conducted both automatically using EndNote as well as manually by study authors. Preliminary title screening was conducted to remove reviews, protocols, conference abstracts and other ineligible study types before remaining studies were exported into Covidence Systematic Review Software (Veritas Health Innovation, Melbourne, Australia, available at http://www.covidence.org).

Using Covidence, titles or abstracts of retrieved studies were screened independently by two study authors (M.S. and C.M.) to identify studies that met the inclusion criteria. Any disagreements were resolved through discussion with an additional reviewer (M.P.). The full texts of potentially eligible studies were then retrieved and independently assessed for eligibility by two review authors (divided between M.S., C.M., M.P., and M.F.‐E.), with any disagreements resolved by a third reviewer.

### 2.4 Data extraction

Data were extracted from all eligible studies independently by study authors (divided between M.S., C.M., M.P., and M.F.‐E.) into a spreadsheet (Microsoft Excel). Extracted information included the author and publication year, animal characteristics, including brain injury model, species, age, and the number of animals included for each outcome. Details of the intervention that were captured included cell type, donor source, dose, the use of immunosuppression, timing and route of administration and comparator. Additionally, outcome assessment details and data were extracted. We have classified all cells derived from embryonic or fetal brain tissue as “fetal tissue derived NSCs” and all neural lineage cells derived from iPSC, ESC, or adult cells as “iPSC‐, ESC‐, or adult tissue derived NPCs.” Any identified discrepancies were resolved through discussion with an additional author. PlotDigitizer (version 2.6.9) was used to quantify the mean and standard deviation or standard error from figures if data were not provided in the text/tables. If relevant data were not available in the published manuscript or supplementary materials, authors were contacted twice, if required. If no response was received, the data were not included.

### 2.5 Risk of bias

Three study authors (divided between M.S., S.M., and M.F.‐E.) independently assessed the risk of bias for each included study using the Systematic Review Centre for Laboratory Animal Experimentation (SYRCLE) risk of bias tool.^27^ SYRCLE assesses selection bias, performance bias, detection bias, attribution bias and reporting bias, reported as “Yes, No or Unclear.” Disagreements were resolved through discussion with additional author/s.

### 2.6 Data synthesis

Review Manager (RevMan) version 5.4 was used to conduct quantitative analysis for primary outcomes when three or more studies assessed brain volume or the same motor or cognitive test. Where studies included an adjuvant or concomitant therapy, only the NSC alone group was used for quantitative analysis. If multiple assessment time points for a single outcome were reported, only the last time point was included in the meta‐analysis. If data were presented as brain volume (tissue remaining), we converted the data to percentage brain infarct volume (tissue loss) to allow assessment by meta‐analysis.

### 2.7 Data analysis

We used a random‐effects, inverse variance model to evaluate the standardized mean difference (SMD) and 95% confidence interval (CI) for all continuous data. The effect of heterogeneity was assessed using the *I* ^*2*^ statistic, with values of 25%, 50%, and 75% considered to be low, moderate, and considerable heterogeneity, respectively. Subgroup analysis was performed where sufficient data were available (two or more studies in each subgroup) to determine potential sources of heterogeneity based on a priori nominated factors including brain injury model, hypoxia length, immunosuppression use, NSC donor species, NSC dose, NSC administration time point post‐injury, and NSC route of administration.

## 3 RESULTS

### 3.1 Study selection

**FIGURE 1:**
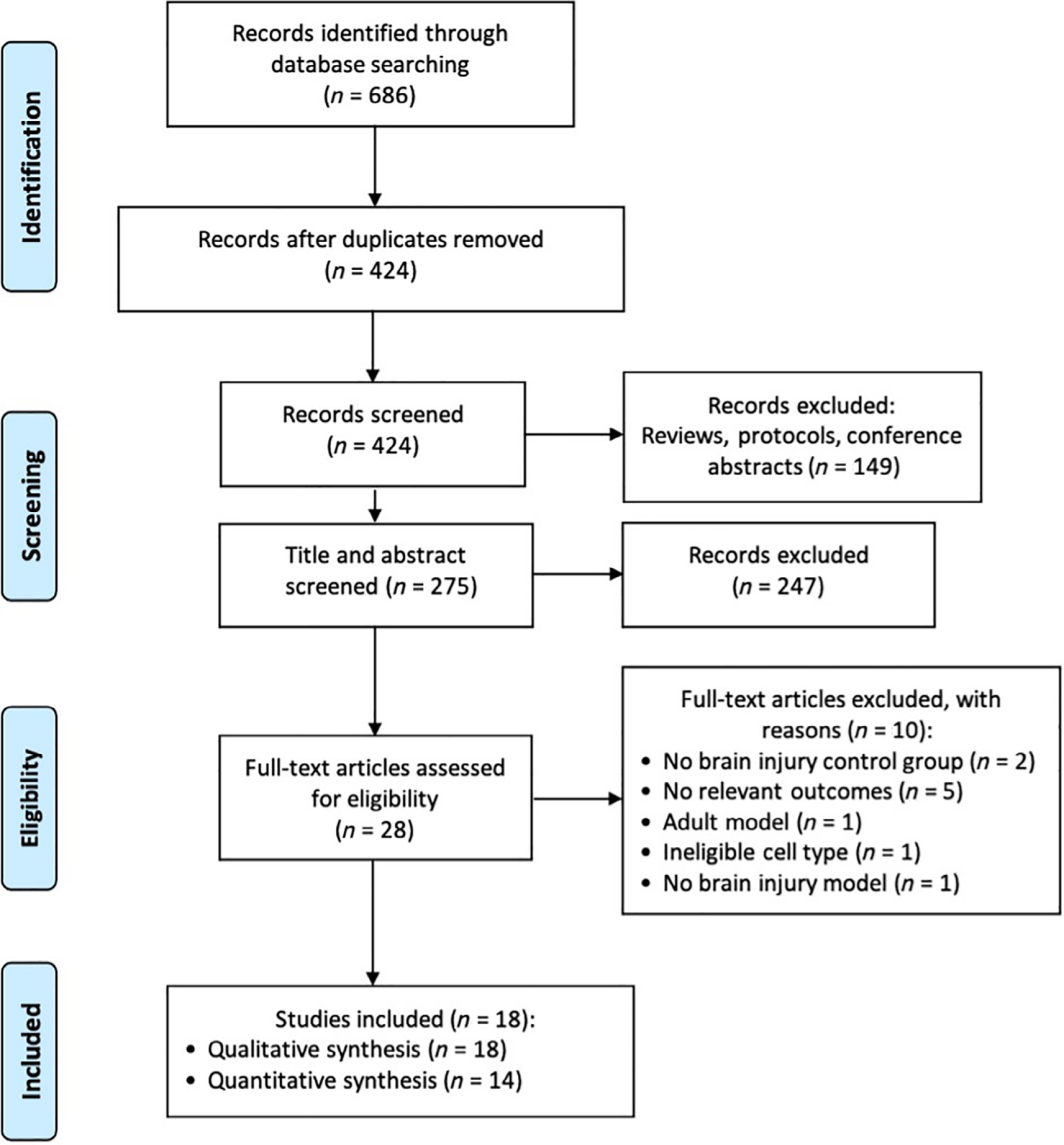
Study selection flow diagram

A total of 686 records were identified following the search procedure shown in Figure 1. After excluding duplicates, 275 studies were screened by title and abstract and 247 studies were subsequently excluded. Full‐text screening for eligibility was performed on 28 studies and, based on the inclusion and exclusion criteria, 18 studies^28^, ^29^, ^30^, ^31^, ^32^, ^33^, ^34^, ^35^, ^36^, ^37^, ^38^, ^39^, ^40^, ^41^, ^42^, ^43^, ^44^, ^45^ were included in this systematic review, with 14 studies included in the meta‐analysis. Updated searches yielded 10 additional records after de‐deduplication. No new records met our inclusion criteria, and thus, all were excluded.

### 3.2 Characteristics of included studies

Characteristics of included studies are summarized in Table 1. All studies were performed in rats (n = 9, 50%) or mice (n = 9, 50%). The brain injury models used were hypoxic ischemic (HI) injury (n = 14, 78%), ischemic injury (n = 2, 11%), excitotoxic injury (n = 1, 6%), and HI plus inflammation injury (n = 1, 6%). During HI injury induction, the length of hypoxia ranged from 20 to 150 minutes. The age at which injury was induced ranged from postnatal day (PND) 2 to PND12.

**TABLE 1:** Characteristics of included studies

Study	Strain and species	Brain injury model	Control for brain injury procedure	Age injury induced	Cell type	Total cells per dose	Cell administration time post‐injury	Administration route and details of location if relevant	Immunosuppression	Comparator
Braccioli 2017 (a)^28^	C57BL/6J mice	HI, right common carotid artery ligation and 10% O_2_ for 45 min	Sham surgery, anesthesia, and incision only	PND9	Mouse fetal tissue NSCs	1 × 10^5^	10 days	Intracerebral injection into the ipsilateral hippocampus	No	Injury + saline
Braccioli 2017 (b)^29^	C57BL/6J mice	HI, right common carotid artery ligation and 10% O_2_ for 45 min	Sham surgery, anesthesia, and incision only	PND9	Mouse fetal tissue NSCs +/− FOXP1 KO	1 × 10^5^	10 days	Intracerebral injection into the ipsilateral hippocampus	No	Injury + saline
Chau 2014^30^	Wistar rats	Focal cerebral ischemic injury, anesthetized with hypothermia and ligation of right middle cerebral artery	Sham surgery, hypothermia, and incision only	PND7	Mouse iPSC‐derived NPCs	4 × 10^5^	7 days	Intracerebral injections at four sites in the peri‐infarct region	No	Injury + media
Comi 2008^31^	CD1 mice	Ischemic injury, right common carotid artery ligation, no hypoxia	Sham surgery, anesthesia, and incision only	PND12	Mouse ESC‐derived NPCs	1 × 10^5^	2 or 7 days	Intracerebral injection into striatum, hemisphere not specified	No	Injury + vehicle^a^
Daadi 2010^32^	SD rats	HI, left common carotid artery ligation and 8% O_2_ for 90 min	No sham control	PND7	Human ESC‐derived NPCs	3 × 10^5^	1 day	Intracerebral injections into three sites of the ipsilateral hemisphere	No	Injury + vehicle^a^
Ji 2015^33^	SD rats	HI, left common carotid artery ligation and 7.8% O_2_ for 120 min	Sham surgery, without ligation nor hypoxia	PND7	Human fetal tissue NSCs	3 × 10^5^	1 day	Intranasal	No	Injury + saline
Kim 2018^34^	SD rats	HI + inflammation, left common carotid artery ligation and 8% O_2_ for 120 min followed by IP injection of LPS	Sham surgery, exposure of the artery without occlusion and vehicle treatment	PND7	Human fetal tissue NSCs differentiated to OPCs	4 × 10^5^ (single dose) OR 1.6 × 10^6^ (repeated doses)	3 days (single dose) OR 3, 10, 20 and 30 days (repeated doses)	Intraventricular injections into four regions, hemisphere not specified	No	Injury + no vehicle
Li 2015^35^	SD rats	HI, left common carotid artery ligation and 8% O_2_ for 150 min	No sham control	PND7	Human fetal tissue NSCs	2.5 × 10^5^	7 days	Intracerebral injection into the ipsilateral ventricle	No	Injury + saline
Rumajogee 2018^36^	C57Bl/6 mice	HI, right common carotid artery ligation and 8% O_2_ for 45 min	Sham surgery, no carotid occlusion was performed but still exposed to hypoxia	PND7	Mouse adult tissue NPCs	2.5 × 10^5^	14 days	Intracerebral injection into the ipsilateral corpus callosum	Yes, cyclosporine	Injury + vehicle^a^
Sato 2008^37^	SD rats	HI, right common carotid artery ligation and 8% O_2_ for 120 min	No sham control	PND7	Rat fetal tissue NSCs +/− chABC	2.5 × 10^5^	1 day	Intraventricular injection into the ipsilateral ventricle	No	Injury + saline
Shin 2018^38^	ICR mice	HI, right common carotid artery ligation and 8% O_2_ for 90 min	Sham surgery, anesthesia, and incision only	PND7	Human fetal tissue NSCs +/− scaffold	9.6 × 10^5^	7 days	Intracerebral injection into the ipsilateral infarct cavity	Yes, cyclosporine	Injury + media
Shinoyama 2013^39^	ICR mice	HI, right carotid artery ligation and 8% O_2_ for 20 min	Sham surgery, opening skin of skull only	PND2	Mouse ESC‐derived NPCs	2 × 10^5^	2 days	Intracerebral injection into the ipsilateral cerebral cortex	No	Injury + no vehicle
Tan 2014^40^	SD rats	HI, left common carotid artery ligation and 8% O_2_ for 120 min	Sham surgery, anesthesia, and incision only	PND7	Rat fetal tissue NSCs +/− VEGF	1 × 10^5^	3 days	Intracerebral injection into the ipsilateral sensorimotor cortex	No	Injury + saline
Titomanlio 2011^41^	WT Swiss mice	Excitotoxic, intracerebral injection of ibotenate into right hemisphere	PBS injection	PND5	Mouse fetal tissue NSCs	3 × 10^5^	4 hours OR 72 hours	Intraventricular injection into the contralateral ventricle	No	Injury + fibroblasts or saline
Wang 2014^42^	C57/BL6 mice	HI, right common carotid artery ligation and 8% O_2_ for 90 min	Anesthesia only	PND7	Mouse fetal tissue NSCs +/− hypothermia	3 × 10^5^	1 day	Intracerebral injection into the ipsilateral ventricle	No	Injury + no vehicle
Yao 2016^43^	SD rats	HI, left carotid artery ligation and 8% O_2_ for 120 min	Sham surgery, anesthesia, and incision only	PND7	Rat fetal tissue NSCs +/− VEGF	1 × 10^5^	3 days	Intracerebral injection into the ipsilateral sensorimotor cortex	No	Injury + saline
Ye 2018^44^	ICR mice	HI, right common carotid artery ligation and 10% O_2_ for 45 min	Sham surgery, anesthesia, and incision only	PND9	Mouse fetal tissue NSCs +/− bFGF	1 × 10^6^	3 days	Intranasal	No	Injury + vehicle^a^
Zheng 2012^45^	SD rats	HI, left common carotid artery ligation and 8% O_2_ for 120 min	Sham surgery, anesthesia, and incision only	PND7	Rat fetal tissue NSCs +/− VEGF	1 × 10^5^	3 days	Intracerebral injection into the ipsilateral somatosensory cortex	No	Injury + saline

Abbreviations: bFGF, basic fibroblast growth factor; chABC, chondroitinase ABC; FOXP1, Forkhead Box P1; HI, hypoxic ischemic; IP, intraperitoneal; iPSCs, induced pluripotent stem cells; KO, knockout; LPS, lipopolysaccharide; NDPs, neurosphere derived precursor cells; NPCs, neural progenitor cells; NSCs, neural stem cells; NSPCs, neural stem progenitor cells; O_2_, oxygen; OPCs, oligodendrocyte progenitor cells; PBS, phosphate buffered saline; PND, postnatal day; SD, Sprague‐Dawley; VEGF, vascular endothelial growth factor; WT, wildtype.

^a^ Vehicle not specified.

The majority of studies used fetal tissue‐derived NSCs (n = 12, 67%), NPCs differentiated from ESCs (n = 3, 17%), NPCs differentiated from iPSCs (n = 1, 6%), adult tissue‐derived NPCs (n = 1, 6%) or fetal tissue NSCs differentiated into OPCs (n = 1, 6%). The cells were derived from either rodent (n = 13, 72%) or human (n = 5, 28%) donors. Some studies used modified donor cells that had upregulation of VEGF (n = 3, 17%), bFGF (n = 1, 6%) or FOXP1 knockout (n = 1, 6%). Cells were administered either directly into the brain via intracerebral injection (n = 13, 72%) or intraventricular injection (n = 3, 17%), or intranasally (n = 2, 11%). The majority of studies that injected cells directly into the brain administered cells into the injured hemisphere (ipsilateral hemisphere) (n = 13/16). In contrast, one study administered cells into the uninjured ventricle (contralateral ventricle) and two studies did not specify. The majority of studies administered a single dose of cells (n = 17, 94%), with one study including a repeated (four times) dose group (6%). The total dose of cells administered ranged from 1 × 10^5^ to 1.6 × 10^6^ cells. The timing of cell administration ranged from 4 hours to 14 days after the induction of brain injury.

Immunosuppression was used in two studies (11%), and both used cyclosporine. Three studies included either a concomitant therapy (hypothermia (n = 1) or chondroitinase ABC (n = 1)) or included an NSC group that was administered on a poly (glycolic acid)‐based scaffold (n = 1). However, as required by our inclusion criteria, all of these studies included an NSC alone group.

### 3.3 Primary outcomes

The results from the primary outcomes for this systematic review are summarized in Table 2.

**TABLE 2:** Summary of primary outcomes of included studies

Study	Intervention	Brain injury (cull time point post‐injury)	Motor function outcomes (time point/s post‐injury)	Cognitive function outcomes (time point/s post‐injury)
Braccioli 2017^28^	NSCs	Infarct size: reduced 56 d	Cylinder test: improved 28, 56 d	
Braccioli 2017^29^	NSCs		Cylinder test: improved 28 d	
	NSCs FOXP1 KO		Cylinder test: NS	
Chau 2014^30^	NPCs		Forelimb placement test: NS	
Comi 2008^31^	NSCs after 2 d	Infarct size: reduced 28 d		
	NSCs after 7 d	Infarct size: NS		
Daadi 2010^32^	NSCs	Infarct size: NS	Cylinder test: improved 28, 31 d Rotarod test: improved 30, 32 d	
Ji 2015^33^	NSCs	Infarct size: reduced 43 d	Gait test: improved 6 d Grid walking test: improved 8 d	Morris water maze: improved 33 d Social choice test: improved 29 d
Kim 2018^34^	OPCs single dose		Cylinder test: improved 23 d Rotarod test: improved 7, 13, 23, 33 d Open field test: improved 13, 23, 33 d	Morris water maze: improved 30 d Passive avoidance test: improved 38 d
	OPCs repeated doses		Cylinder test: improved 23 d Rotarod test: improved 13, 23, 33 d Open field test: improved 13, 23, 33 d	Morris water maze: improved 30 d Passive avoidance test: improved 38 d
Li 2015^35^	NSCs			Morris water maze: improved 29 d
Rumajogee 2018^36^	NPCs	Hemisphere size: NS	Cylinder test: improved 42, 49, 56, 63, 77 d Cat walk test: improved 21, 28, 35, 42, 49, 56, 63, 77 d	
Sato 2008^37^	NSPCs	Hemisphere size: NS		
	NSPCs + chABC	Hemisphere size: reduced 9 d		
Shin 2018^38^	NPCs	Infarct size: NS	Rotarod test: NS	
	NPCs + scaffold	Infarct size: reduced 63 d	Rotarod test: improved 84 d	
Shinoyama 2013^39^	NPCs		Rotarod test: improved 23 d Beam walking test: improved 23 d	
Tan 2014^40^	NSCs		Holding test: improved 23 d	Radial arm test: improved 23 d
	NSCs + VEGF		Holding test: improved 23 d	Radial arm test: improved 23 d
Titomanlio 2011^41^	NDPs after 4 h	Gray and white matter infarct size: reduced 5 d	Open field: NS	Novel object recognition test: improved 16, 35 d
	NDPs after 72 h		Open field: NS	Novel object recognition test: improved 16 d
Wang 2014^42^	NSCs	Infarct size: NS	Cylinder test: NS Rotarod test: NS	Morris water maze: NS
	NSCs + hypothermia	Infarct size: reduced 7, 14, 28 d	Cylinder test: NS Rotarod test: improved 22, 29, 36 d	Morris water maze: improved 57, 169 d
Yao 2016^43^	NSCs		Attitudinal reflex test: improved 27 d	Radial arm test: improved 27 d
	NSCs + VEGF		Attitudinal reflex test: improved 27 d	Radial arm test: improved 27 d
Ye 2018^44^	NSCs	MAP‐2 volume loss: reduced 35 d	Cylinder test: improved 21, 28, 35 d Adhesive removal test: improved 35 d	
	NSCs + bFGF	MAP‐2 volume loss: reduced 35 d	Cylinder test: improved 21, 28, 35 d Adhesive removal test: improved 35 d	
Zheng 2012^45^	NSCs		Foot fault test: improved 17 d	*T* test: improved 17 d
	NSCs + VEGF		Foot fault test: improved 17 d	*T* test: improved 17 d

*Note*: Empty cells = did not measure this outcome, improved/reduced = significant improvement or reduction following NSC treatment, compared with the injured control (*P* < .05).

Abbreviations: bFGF, basic fibroblast growth factor; chABC, chondroitinase ABC; FOXP1, Forkhead Box P1; KO, knockout; MAP‐2, microtubule‐associated protein 2; NDPs, neurosphere derived precursor cells; NPCs, neural progenitor cells; NS, not significant; NSCs, neural stem cells; NSPCs, neural stem progenitor cells; OPCs, oligodendrocyte progenitor cells; VEGF, vascular endothelial growth fact.

#### 3.3.1 Effect of NSCs on brain infarct volume

Ten of the 18 included studies assessed brain infarct volume using either infarct volume (n = 7), injured hemisphere volume (n = 2) or gray/white matter loss (n = 1), measured at time points between 5 and 77 days post‐injury. Five studies showed that treatment with NSCs led to a reduction in brain infarct size, and five showed no significant difference compared with injured controls (Table 2). One study,^31^ which looked at early (2 days) vs delayed (7 days) administration, showed that infarct size was reduced following administration at 2 days post‐injury but not at 7 days.

**FIGURE 2:**
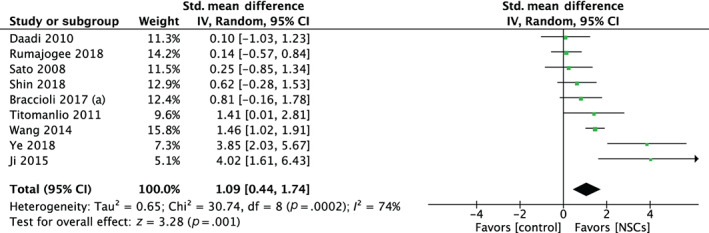
Forest plot demonstrating the effect of neural stem cells compared with controls on brain infarct volume

After excluding one study that used an incomparable unit of measurement (median and interquartile range),^31^ the meta‐analysis revealed that NSCs significantly decreased infarct volume by an SMD of 1.09 (95% CI: 0.44, 1.74, *P* = .001; X^2^ = 30.74, *I* ^2^ = 74%, *P* = .0002), with effect sizes ranging from 0.10 to 4.02 when compared with injured controls (Figure 2).

#### 3.3.2 Effect of NSCs on motor function

A total of 15 of the 18 studies assessed motor function, with 11 showing that NSCs significantly improved motor function compared with injured controls on at least one motor outcome and at one or more time point/s (Table 2). In contrast, four studies failed to show any significant between‐group difference for any motor function assessment. A range of motor assessments were used across the studies, and many studies assessed motor function via two or more different motor assessments. Interestingly, most studies that used multiple assessments (n = 7/8) showed either a statistically significant improvement or an insignificant result across all of their chosen motor assessments. The most common assessment was impaired forelimb use, measured by the cylinder test (n = 7) or forelimb placement test (n = 1) between 7 and 77 days post‐injury, followed by the rotarod test (n = 5) similarly measured between 7 and 84 days post‐injury. One study included a repeat dose (four doses) group and a single dose group.^34^ This study showed that both single and repeated doses of OPCs improved motor function via the cylinder test, rotarod test and open field test. There were no significant differences between the two groups, except for a slightly earlier onset of motor improvement in the single dose group.

**FIGURE 3:**
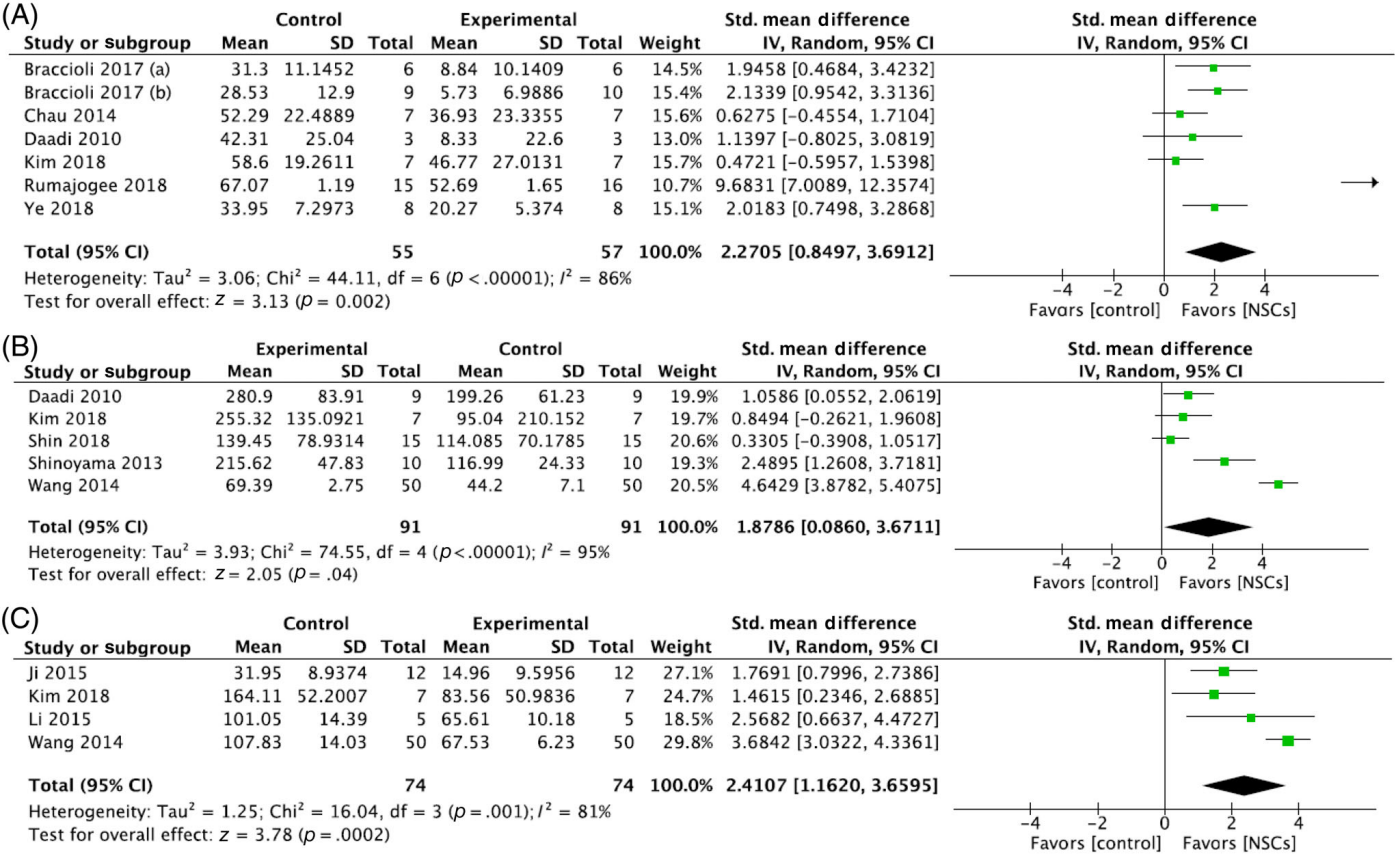
Forest plot demonstrating the effect of neural stem cells compared with controls on (A) impaired forelimb use, (B) rotarod test performance, and (C) Morris water maze test performance

For impaired forelimb use, six studies showed that NSC administration significantly improved the use of the impaired forelimb, and two showed no improvement (Table 2). After exclusion of one study that used an incomparable unit of measurement (data expressed as frequency rather than percentage),^42^ a meta‐analysis showed that NSCs significantly improved impaired forelimb use following brain injury by an SMD of 2.27 (95% CI: 0.85, 3.69, *P* = .002; X^2^ = 44.11, *I* ^2^ = 86%, *P* < .00001) with effect sizes ranging from 0.47 to 9.68 (Figure 3).

Three studies showed a significant improvement following NSC administration for rotarod performance, and two studies showed no improvement compared with injured controls. Meta‐analysis of the five studies demonstrated that NSCs significantly improved performance in the rotarod test following brain injury by an SMD of 1.88 (95% CI: 0.09, 3.67, *P* = .04; X^2^ = 74.55, *I* ^2^ = 95%, *P* < .00001) with effect sizes ranged from 0.33 to 4.64 (Figure 3).

#### 3.3.3 Effect of NSCs on cognitive function

Less than half of the included studies assessed cognitive function (n = 8/18). Of these, seven studies showed improvement on at least one cognitive test, at one or more time points, with only one study showing no improvement (Table 2). The most commonly reported cognitive outcome assessment was the Morris water maze test (n = 4 studies), assessed at time points between 29 and 169 days post‐injury. One study included a repeat dose (four doses) group^34^ and a single dose group. This study showed that both single and repeated doses of OPCs improved cognitive function via the Morris water maze and passive avoidance tests, and there were no significant differences between the two groups.

Three studies showed that NSCs significantly improved the function on the Morris water maze test (Table 2). From the meta‐analysis, NSCs significantly improved performance compared with injured controls on the Morris water maze test following brain injury by an SMD of 2.41 (CI: 1.16, 3.66, *P* = .0002; X^2^ = 16.04, *I* ^2^ = 81%, *P* = .0002) with effect sizes ranging from 1.46 to 3.68 (Figure 3).

#### 3.3.4 Subgroup analysis

Subgroup analysis of relevant parameters and their contribution to outcome heterogeneity is presented in Supplementary Figures S1 and S2. Due to the limited number of studies, we were unable to run analyses for NSC donor species, NSC dose, brain injury model and hypoxia duration. When a sufficient number of studies were available, subgroup analysis of brain infarct size showed no difference in effect size between cell administration time point (*P* = .17) or species of the donor NSCs (*P* = .67) (Supplementary Figure S1a,c). While studies that did not use immunosuppression had a larger reduction in infarct size compared with those that did (*P* = .03), this result should be interpreted with caution due to the small number of studies (n = 2) that used immunosuppression (Supplementary Figure S1b). Moreover, high heterogeneity was noted even within the group that did not use immunosuppression (*I* ^2^ = 72%). Additionally, studies that administered NSCs via intranasal delivery had a larger reduction in infarct size compared with both intracerebral and intraventricular delivery (*P* < .0001, *P* = .0007, respectively) (Supplementary Figure S1d), but this result should be interpreted with caution since only two studies delivered NSCs intranasally and high heterogeneity was noted (*I* ^2^ = 74%). For impaired forelimb use, the cell administration time point did not affect the effect size (*P* = .17) (Supplementary Figure S2). No subgroup analyses could be conducted for cognitive outcomes due to the low number of studies that reported the same cognitive assessments.

### 3.4 Secondary outcomes

#### 3.4.1 NSC survival, migration, differentiation, and neuroinflammation

The secondary outcomes of interest are summarized in Table 3. Cell survival was always assessed by brain histology after animals were culled (time points ranging from 5 to 133 days). All studies that assessed NSC survival (n = 14) detected donor cells within the brain. Seven studies showed evidence of NSC migration, with a variety of methods used to assess migration. One study reported NSC migration toward the site of injury, whereas implanted control cells (fibroblasts) remained at the injection site.^41^ Differentiation into neurons, oligodendrocytes and astrocytes was assessed via morphology or a range of immunohistochemical markers. In total, of the studies that analyzed differentiation (n = 14), all studies (14/14) found that NSCs differentiated into neurons. Only four studies investigated oligodendrocyte differentiation and all four successfully detected oligodendrocytes. In contrast, only seven out of nine studies confirmed astrocyte differentiation. Three studies showed that treatment with NSCs decreased brain inflammation (anti‐inflammatory response) assessed by either the number of microglia and macrophages (Iba‐1) or IL‐1β expression. Contrastingly, one study found that NSC treatment increased the number of microglia in the striatum, indicating a pro‐inflammatory response against transplanted NSCs.

**TABLE 3:** Summary of secondary outcomes of included studies

	Survival	Migration	Differentiation^a^			Neuroinflammation
Study	Last time point cells detected^b^	Evidence of cell migration?	Neuron	Astrocyte	Oligodendrocyte	Effect on inflammation
Braccioli 2017^28^	5 d	Yes	✓ Doublecortin	× GFAP	—	Anti‐inflamatory (Iba‐1)
Braccioli 2017^29^	5 d	—	✓ Doublecortin	—	—	—
Chau 2014^30^	7 d	—	✓ NeuN, neurofilament	✓ GFAP	—	—
Comi 2008^31^	26 d	—	✓ Morphology	—	—	—
Daadi 2010^32^	28 d	—	✓ NeuN, TuJ1+, doublecortin, GAD	✓ GFAP	—	Pro‐inflammatory (Iba‐1)
Ji 2015^33^	42 d	Yes	✓ NeuN	✓ GFAP	—	Anti‐inflammatory (IL‐1β)
Kim 2018^34^	35 d	—	NA^c^	NA^c^	✓ Olig2, MBP	—
Li 2015^35^	28 d	—	✓ NSE	—	—	—
Rumajogee 2018^36^	133 d	Yes	✓ NeuN, doublecortin	✓ GFAP	✓ Olig2	—
Sato 2008^37^	7 d	Yes	✓ MAP‐2	—	—	—
Shin 2018^38^	84 d	—	✓ Neurofilament	✓ GFAP	—	Anti‐inflammatory (Iba‐1)
Shinoyama 2013^39^	21 d	Yes	✓ NeuN, Ctip2	—	—	—
Tan 2014^40^	—	—	—	—	—	—
Titomanlio 2011^41^	37 d	Yes	✓ MAP2, NeuN	× GFAP	✓ NG2, MBP	—
Wang 2014^42^	28 d	—	✓ NeuN	✓ GFAP	✓ CNPase	—
Yao 2016^43^	—	—	—	—	—	—
Ye 2018^44^	—	Yes	✓ NeuN	✓ GFAP	—	—
Zheng 2012^45^	—	—	—	—	—	—

*Note*: (—) = not assessed, ✓ = yes, x = no differentiation

Abbreviations: CNPase, 2′,3′‐cyclic‐nucleotide 3′‐phosphodiesterase; Ctip2, chicken ovalbumin upstream promotor transcription factor 2; GAD, glutamic acid decarboxylase; GFAP glial fibrillary acidic protein; Iba‐1, ionized calcium binding adaptor protein 1; IL‐1β, interleukin 1 beta; Inflam, inflammatory; MAP‐2, microtubule‐associated protein 2; MBP, myelin basic protein; NA, not applicable; NeuN, neuronal nuclei; NG2, nerve/glial antigen 2; NSE, neuron‐specific enolase; Olig2, oligodendrocyte transcription factor 2; TuJ1+, neuron‐specific class III β‐tubulin.

^a^ Differentitation markers.

^b^ Time point post NSC administration.

^c^ This study used an oligodendrocyte progenitor cell, and therefore we would not expect this cell type to differentiate into neurons or astrocytes.

### 3.5 Modifications and concomitant therapies

Some studies included in this systematic review also tested the efficacy of modified donor cells, concomitant therapies or cell scaffolding, and their effect on the primary outcomes are summarized in Table 2. Notably, three studies showed that the inclusion of additional interventions (hypothermia,^42^ chondroitinase ABC,^37^ or scaffolding^38^) led to a significant reduction in infarct size compared with NSCs alone. However, one study that assessed the added effect of bFGF overexpressing NSCs^44^ showed no difference. For motor function, applying a scaffold or adding hypothermia produced a significant improvement in rotarod performance compared with NSCs alone. However, bFGF or VEGF overexpression did not affect a variety of motor outcomes. Interestingly, the knockdown of FOXP1 reduced the effectiveness of NSCs measured by the cylinder test. Similarly, for cognitive function, the addition of hypothermia improved Morris water maze performance; however, overexpression of VEGF did not improve cognition measured by the radial arm test or *T* test.

### 3.6 Risk of bias assessment

**FIGURE 4:**
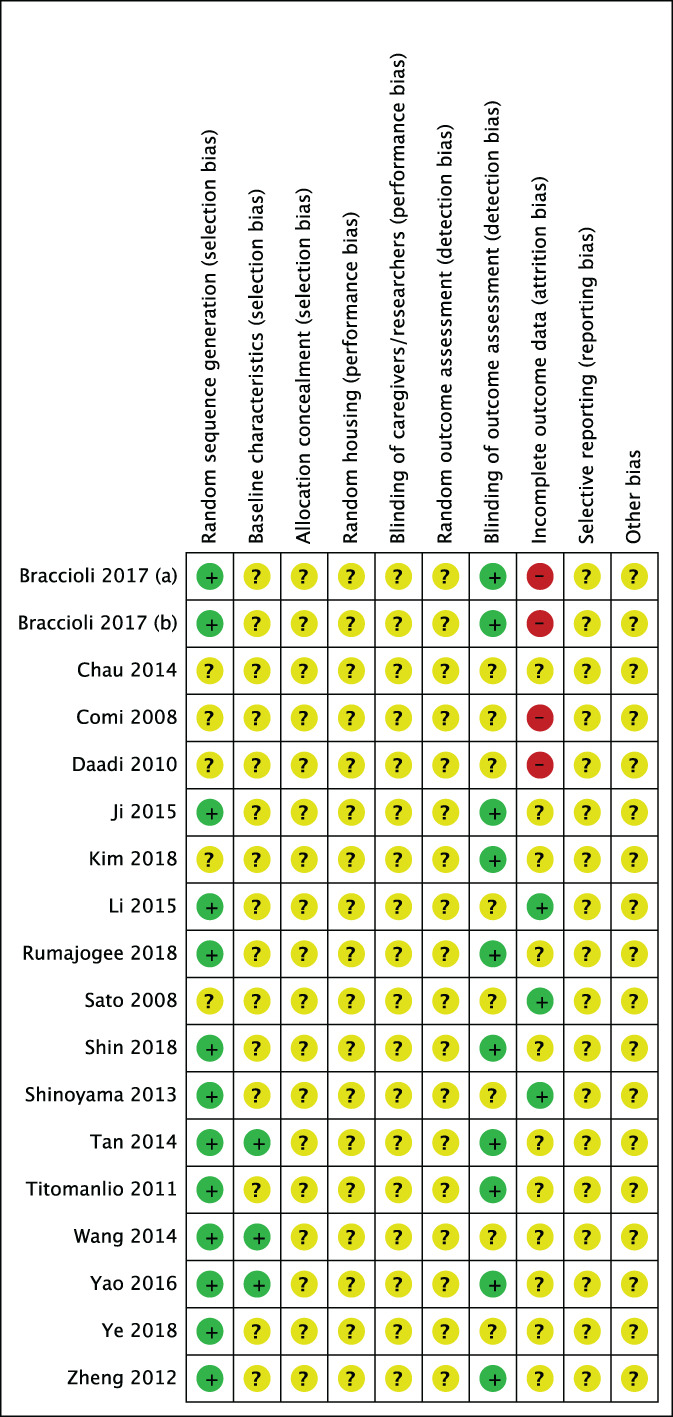
Risk of bias of the included studies: + = low risk of bias,? = unclear risk of bias, and – = high risk of bias

The risk of bias across included studies is summarized in Figure 4. No studies were judged to have a low risk of bias across all domains. Selection bias was low in most studies when examining randomization, but few studies described baseline characteristics of included animals. Additionally, no studies specifically described the method of random sequence generation. Across all studies, allocation concealment, random housing, blinding of caregivers, and random outcome assessment was not described. Approximately half (n = 8/18) of the included studies described blinding during outcome assessment. Attrition bias was variable across studies, with a high risk of bias assigned to four studies that did not account for the decrease in animal numbers reported between methods and results. Three studies had a low risk of attrition bias, and the remainder were unclear. Selective reporting bias was unclear across all studies, as published protocols were not available. We did not identify any other sources of bias.

## 4 DISCUSSION

There are limited treatments available for perinatal brain injury, and NSCs show strong neuroregenerative potential, with mounting preclinical data. From 18 studies identified, this meta‐analysis showed that NSC administration significantly improved motor (forelimb use, rotarod test) and cognitive outcomes (Morris water maze test) and neuropathology (infarct size) following perinatal brain injury. Additionally, NSCs commonly demonstrated functional ability to migrate, survive long term, differentiate into the three main cell types of the brain, and reduce inflammation. Although these were not the primary outcomes of interest, neurohistopathological findings are important in determining the biological mechanisms that could be driving behavioral improvements, otherwise difficult to elucidate in humans.

### 4.1 Primary outcomes

This systematic review aimed to measure the efficacy of NSC administration for perinatal brain injury to identify the areas of research with insufficient data and key knowledge gaps. Primary outcomes assessed in this systematic review were brain infarct size and motor and cognitive outcomes, which have clinical relevance since it is well known that perinatal brain injury often leads to motor deficits such as cerebral palsy,^46^ cognitive deficits, and neurodevelopmental delays.^47^ There was variability in the behavioral outcomes assessed across studies, highlighting the need to standardize outcomes, and ensure that they closely measure relevant clinical outcomes of perinatal brain injury. Standardization of outcomes and use of measures that hold clinical relevance would provide more power to meta‐analyses, so that robust efficacy data can progress NSC therapy along the translational research pipeline. We have found that motor function testing predominantly used impaired forelimb and rotarod tests, and given that 95% of included studies used a unilateral brain injury model that more closely mimics hemiplegic cerebral palsy,^48^ these tests were clinically relevant and should be used more widely. In addition, the Morris water maze was the most common cognitive test used, which assesses learning and memory deficits that can occur in humans after perinatal brain injury.^47^ Given that the key benefit of NSCs is the potential to repair the injured brain,^49^ the effect of these cells on infarct size was also an important outcome. Overall, our meta‐analysis showed that NSCs significantly decreased brain infarct volume and improved motor and cognitive outcomes, indicating that NSCs are effective in reducing the severity of perinatal brain injury and alleviating functional deficits. Our results are consistent with meta‐analyses of preclinical models of adult stroke.^20^, ^22^ Both of these adult stroke meta‐analyses reported a SMD of <1 in the cylinder test (impaired forelimb use), whereas we had a comparatively larger SMD of 2.27. SMD for infarct size were similar to that seen in adult stroke models. Additionally, the SMD reported for the rotarod test was 1.88, which was similar to the SMD reported in the adult stroke meta‐analyses. The NSC field is more advanced in the area of adult diseases and phase I trials have shown safety in adults following stroke^50^ and has now moved to phase II clinical trials.^51^ Interestingly, two phase I clinical trials have been completed in pediatric neuronal ceroid lipofuscinosis and Pelizaeus‐Merzbacher disease and have shown safety^52^, ^53^ and support the further investigation of NSCs for pediatric neurological conditions.

### 4.2 Secondary outcomes

The secondary outcomes of interest in this systematic review included the potential of NSC differentiation, survival, migration, and the effect of NSC therapy on neuroinflammation. Neuroregeneration is a proposed mechanism of action of NSC therapy,^49^ which likely relies on NSC survival and differentiation into neural cells within the damaged brain. Encouragingly, in most studies that investigated NSC differentiation, NSCs were able to differentiate into neurons. This is consistent with studies demonstrating that NSCs first differentiate into neurons followed by glial cells.^54^ Differentiation into astrocytes was observed, but not as commonly, and oligodendrocyte differentiation was generally observed at later cull time points (>28 days of transplantation). Additionally, NSCs were present within the brain for up to 133 days after administration, which suggests these cells may have the ability engraft long‐term in the neonatal brain. Studies in rodent models of spinal cord injury have shown that long‐term engraftment of NSCs may be a necessary process to promote functional improvement, since selective ablation of exogenous NSCs reversed locomotor recovery.^55^ Consequently, engraftment is likely necessary to elicit improvements in clinical outcomes following NSC administration in the injured perinatal brain and encouragingly this review has shown that NSC survival is possible.

Interestingly, while one of the main proposed mechanisms of action of NSCs is their immunomodulatory capacity,^56^ few studies investigated this outcome. From these few studies, the majority demonstrated anti‐inflammatory actions; however, one study indicated that NSC transplantation exacerbated inflammation. More research is warranted to reach consensus on the anticipated effect of NSC administration on the neonatal neuroinflammatory response, especially since there is a growing body of research characterizing the NSC secretome and highlighting the neurotrophic action of NSCs in various diseases of the central nervous system.^57^ Other therapeutic stem cells widely explored to treat perinatal brain injury include MSCs and UCBCs which have well described immunomodulatory capacity, but a comparison of cell sources was beyond the scope of this study. A direct comparison between NSCs, MSCs, and UCBCs would address which cell type may be most beneficial to reduce inflammation.

### 4.3 The effect of concomitant therapy on NSC efficacy

A number of studies used concomitant therapies or NSC modifications with the objective to enhance the efficacy of NSC treatment. Due to the fact that each of the modifications have different underlying mechanisms of action, we could unfortunately not include these groups in the meta‐analyses as it would not have been appropriate to combine them as one group, and our analyses would have been underpowered if they had been analyzed separately. Although these groups were not included in the meta‐analysis, the studies that did included the use of concomitant therapies with NSCs demonstrated improved outcomes to a greater extent compared with NSC treatment alone. In particular, the addition of hypothermia, scaffolding and chondroitinase ABC were more efficacious. Interestingly, each of these studies failed to show a significant effect when NSCs were given alone, which contradicts the overall conclusions from our meta‐analysis but may indicate that concomitant therapies with NSC transplantation are superior. Additionally, NSC modification via transgenic upregulation of growth factors (eg, VEGF and bFGF) appears to enhance efficacy beyond that seen for unmodified NSCs. Given the promising evidence that transgenic upregulation of growth factors may increase efficacy, further investigation is warranted to understand what modifications are the most beneficial and more research is needed to determine the effect NSCs may have in combination with hypothermia, which is already used to treat HIE.

### 4.4 Knowledge gaps identified

Conducting this systematic review and meta‐analysis enabled us to identify knowledge gaps that need to be addressed to progress this area of research. First, the mechanisms by which NSCs can elicit their beneficial effects remains unclear but likely includes cell replacement, immunomodulation, neurotrophic action, the acceleration of endogenous recovery, or a combination of these mechanisms.^58^ If engraftment is required for brain regeneration, determining the interplay between the number of engrafted NSCs and efficacy will need to be established. In addition, the fate of these engrafted cells needs to be further investigated beyond identifying that a few cells can differentiate. This will include information such as the percentage of engrafted cells that differentiate and what cell type they become, that is neurons, oligodendrocytes, or astrocytes.

Engraftment is likely dependent on the immune response (or lack thereof) to NSCs by the host immune system. Therefore, the role of immunosuppression, particularly as we translate preclinical research, needs to be considered. It has been shown that transplanted NSC are recognized by the immune system and induce an immune response,^59^ and it has been shown that immunosuppression is required for long‐term engraftment in adult neurological animal models.^60^, ^61^ Additionally, in vivo studies have shown that inflammatory mediators increase MHC class I and II antigen^62^ expression.^51^ Therefore the pro‐inflammatory environment following neonatal brain injury could increase the likelihood of NSC rejection by the host's adaptive immune system. Only two of 18 studies identified in this review used immunosuppression in conjunction with NSC administration. Our subgroup analysis found that no immunosuppression reduced infarct size to a greater extent than those that used immunosuppression. However, the results of this analysis should be interpreted with caution, since only two studies with high heterogeneity were included in the immunosuppression group, and none of these studies directly compared the effect of immunosuppression on long‐term NSC engraftment. The naivety of the neonatal immune system^63^ may be why immunosuppression was omitted in most studies, but further research is required to draw robust conclusions about its necessity. A study that directly compares NSC administration with and without immunosuppression that assesses NSC engraftment in addition to functional outcomes would help to answer this critical research question.

Across all preclinical research to date, we did not identify any studies performed in large animals, which have played a role in the translation of NSC to clinical trials for adult stroke. Nonhuman primate models of adult stroke have shown that NSCs can survive, differentiate, and were tolerated,^64^, ^65^ which has supported the progression to human clinical trials. It is likely that large animal models are a necessary step before human studies, as 99% of neuroregenerative therapies tested solely in rodents have failed to show any benefit in humans.^66^ To prevent this downfall, large animal models with comparable brain size to humans, such as sheep or nonhuman primates should be performed as the next step in the research pipeline.

Other knowledge gaps identified include the lack of direct comparison of the route, dose, source, and timing of NSC administration. Firstly, subgroup analysis found that intranasal delivery was more effective at reducing infarct size compared with both intracerebral and intraventricular delivery of NSCs. However, these results must be interpreted with caution since only two studies administered NSCs intranasally and high heterogeneity was noted. While most studies utilized either intracerebral or intraventricular delivery, further research into less invasive methods including intranasal delivery should be pursued and directly compared with other delivery methods. The variability in timing of NSC administration across studies prevented us from determining the most efficacious time point. Timing is important, since the inflammatory environment changes throughout the course of injury^67^ and may effect the action of NSC treatment. A study in adult mice found that the timing of NSC transplant determines the phenotypic fate of donor cells to either neurons (later transplant) or astrocytes (earlier transplant).^68^ Additionally, previous reviews in adult models have shown that allogeneic NSC transplant causes a more significant reduction in lesion size than xenogeneic transplant,^20^, ^22^ therefore the adaptive immune system could be hindering the beneficial action of NSCs due to rejection. In future, the source (allogeneic or xenogeneic) of NSCs and the use of immunosuppression should be a consideration in the design of preclinical studies. Some studies used NSCs which carry ethical considerations, particularly NSCs derived from fetal sources, including cells isolated directly from fetal brain tissue, and ESC‐differentiated cells. This ethical barrier could be removed altogether by finding an equally efficacious NSC sourced from iPSCs. Notably, only one study identified in this systematic review administered iPSC‐NSCs. Consequently, more research into this cell type is warranted and it is imperative to directly compare efficacy of the various cell sources.

### 4.5 Limitations

In this systematic review, we have specifically focused on the efficacy of NSCs for improving perinatal brain injury. We acknowledge that this is a limitation of the study and there are a number of other stem cell types that are being pursued for perinatal brain injury and have shown much promise.^15^, ^69^, ^70^ In addition, there is interest in stem cell sources that possess the two‐pronged mechanism of neural differentiation and growth factor secretion similar to NSCs, and these include ESCs and iPSCs. However, we are not aware of any studies that have tested the efficacy of undifferentiated cells in preclinical models of perinatal brain injury. It will be important for the field moving forward that head‐to‐head comparison studies of different stem cell types are performed so we can determine the optimal cell type to treat perinatal brain injury. Another limitation of this study was the meta‐analyses were limited by the small number of studies and the high heterogeneity across outcomes, especially the behavioral assessments. Additionally, we were unable to determine the source of heterogeneity, subgroup analysis was limited and publication bias could not be investigated, due to the small number of studies, variation in study design, and intervention characteristics discussed above.

Through our risk of bias assessment, it was identified that there was minimal reporting across all domains, limiting the conclusions drawn from this meta‐analysis. This weakness is widely recognized in preclinical animal research^71^, ^72^ and has likely contributed to the high risk of bias identified in this systematic review. The common areas of bias we identified included selection bias, including under reporting of allocation concealment and baseline characteristics of animals before the study, as well as attrition bias, with some studies having incomplete numbers in their outcome data and not accounting for the loss of animals from outcomes. While it is possible that studies had a low risk of bias in their study design and simply did not report clearly within the domains, especially due to strict word limits for publication, significant concerns to the validity of studies remain. This meta‐analysis highlights the need to for preclinical scientists to rigorously design and report methodology and refer to the SYRCLE and ARRIVE guidelines^27^, ^73^ when publishing to allow future systematic reviews to rigorously interrogate preclinical research.

### 4.6 Conclusions and future directions

The limited treatment options for perinatal brain injury and the subsequent life‐long burden defines an urgent need to develop neuroregenerative treatments. From preclinical research to date, all performed in rodents, we show that NSC administration is an efficacious treatment for perinatal brain injury across neuropathological, motor, and cognitive domains. Before the commencement of clinical trials testing the efficacy of NSC transplantation for perinatal brain injury, we have identified important future directions that preclinical research needs to address first. Knowledge gaps identified include the lack of direct comparison of the route, dose, source, and timing of NSC administration in addition to the standardization of clinically‐relevant behavioral outcomes. Studies in large animal studies are necessary to show the effectiveness of NSC transplantation for perinatal brain injury and further investigation is required into whether immunosuppression is necessary.

## CONFLICT OF INTEREST

M.P. declared employment and research funding from the Cerebral Palsy Alliance Research Foundation. The other authors declared no potential conflicts of interest.

## AUTHOR CONTRIBUTIONS

M.J.S.: conception and design, literature searching, data extraction, risk of bias assessment, data synthesis and analysis, manuscript writing and editing; M.C.B.P.: conception and design, literature searching, data extraction, manuscript writing; M.C.F. and G.J.: conception and design, manuscript editing; S.L.M.: conception and design, risk of bias assessment, manuscript editing; M.F.‐E.: conception and design, literature searching, data extraction, risk of bias assessment, manuscript editing; C.A.M.: conception and design, literature searching, data extraction, data synthesis and analysis, manuscript editing.

## Supporting information


**Figure S1** Subgroup meta‐analysis of brain infarct volume. Forest plots show the effect size of (a) neural stem cell administration time point post‐injury, (b) the use of immunosuppression drugs, (c) neural stem cell source, and (d) neural stem cell administration route.

**Figure S2** A Forest plot of the subgroup meta‐analysis for impaired forelimb use, showing the effect size across two windows of NSC administration time points post‐injury.

## Data Availability

All supporting data and datasets generated for this study are available on request to the corresponding author.
