# Supplementary figures and images for: Neural stem cell treatment for perinatal brain injury: A systematic review and meta‐analysis of preclinical studies
Source: Stem Cells Transl Med. 2021 Sep 20;10(12):1621–36. doi: 10.1002/sctm.21-0243 (PMC8641092; doi:10.1002/sctm.21-0243)

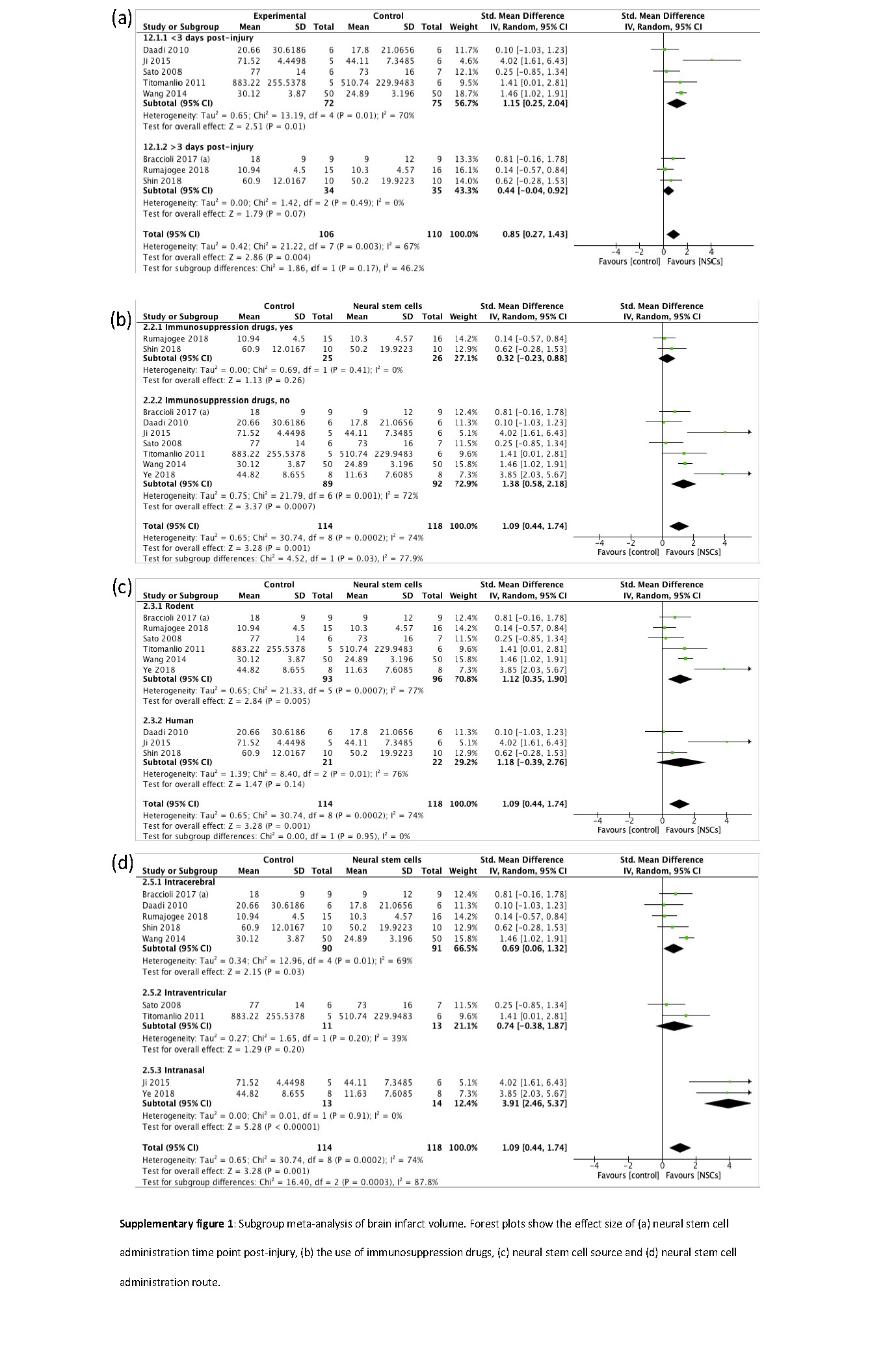

Supplement: Supplementary file 1 — Figure S1 Subgroup meta‐analysis of brain infarct volume. Forest plots show the effect size of (a) neural stem cell administration time point post‐injury, (b) the use of immunosuppression drugs, (c) neural stem cell source, and (d) neural stem cell administration route. [file SCT3-10-1621-s002.jpg]

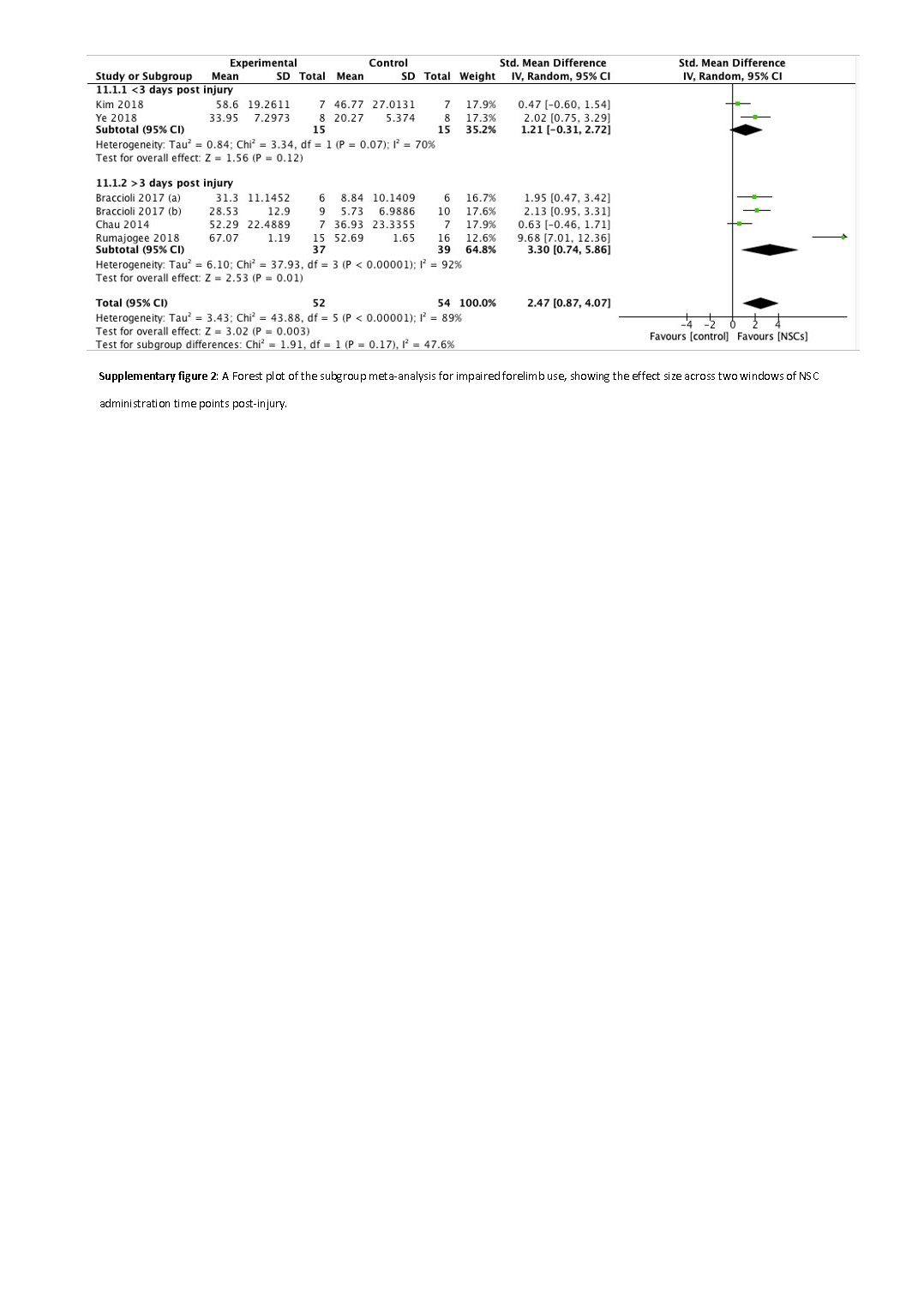

Supplement: Supplementary file 2 — Figure S2 A Forest plot of the subgroup meta‐analysis for impaired forelimb use, showing the effect size across two windows of NSC administration time points post‐injury. [file SCT3-10-1621-s001.jpg]
